# Elevated Fasting Plasma Glucose Is Associated With an Increased Risk of MCI: A Community-Based Cross-Sectional Study

**DOI:** 10.3389/fendo.2021.739257

**Published:** 2021-11-16

**Authors:** Wei Li, Ling Yue, Lin Sun, Shifu Xiao

**Affiliations:** ^1^ Department of Geriatric Psychiatry, Shanghai Mental Health Center, Shanghai Jiao Tong University School of Medicine, Shanghai, China; ^2^ Alzheimer’s Disease and Related Disorders Center, Shanghai Jiao Tong University, Shanghai, China

**Keywords:** fasting plasma glucose, MCI, cross-sectional, elderly, Chinese

## Abstract

**Background:**

Mild cognitive impairment (MCI) is a transitional state between normal elderly people and dementia, with a higher risk of dementia transition. The primary purpose of the current study was to investigate whether routine blood and blood biochemical markers could be used to predict the onset of MCI.

**Methods:**

Data was obtained from the cohort study on brain health of the elderly in Shanghai. A total of 1015 community elders were included in the current study. Based on clinical evaluation and the scores of Montreal Cognitive Assessment (MoCA), these participants were divided into the MCI (n=444) and cognitively normal groups (n=571). Then we tested their fasting blood routine and blood biochemical indexes, and collected their general demographic data by using a standard questionnaire.

**Results:**

By using binary logistic regression analysis and the ROC curve, we found that elevated fasting plasma glucose (p=0.025, OR=1.118, OR=1.014-1.233) was a risk factor for MCI.

**Conclusions:**

Elevated fasting blood glucose may be a risk factor for mild cognitive impairment, but the above conclusions need to be verified by longitudinal studies.

## 1 Introduction

The term of mild cognitive impairment (MCI) was introduced in the late 1980s by Reisberg and colleagues, which represents a state of cognitive function between normal aging and dementia (1). Individuals with MCI often complain about their memory and show mild cognitive deficits on objective cognitive tests, but their cognitive impairment is not severe enough to qualify for dementia (2). However, these MCI individuals (10%-15%) generally have a ten-fold greater risk of progression to dementia than the general population (1-2%), suggesting that MCI is a prodrome stage of dementia (3). Although dementia is irreversible, MCI can be reversed to normal or maintained in a relatively stable state for a long time. Therefore, early identification of risk or protective factors affecting the progression of MCI is extremely important (4).

Previous studies have shown that depression, sex, years after menopause, daily vegetable intake, hormonal replacement, and hypertension can promote the transition from normal elderly to MCI (5–8). Similarly, certain indicators in blood also have a predictive effect. For example, Bingying Du et al. found that the ratio of glycogen Synthase Kinase (GSK)-3β to brain-derived Neurotrophic Factor (BDNF) was strongly related to MCI (9). Simon J Furney et al. found that biochemical markers of inflammation were better predictors of MCI conversion than APOE genotype or clinical measures (10). What’s more, Rosebud O Roberts et al. found that high C-reactive protein was a risk factor for non-amnestic mild cognitive impairment (11).

A systematic review and meta-analysis of 144 prospective studies showed that diabetes, even prediabetes and changes in diabetes-related biochemical markers could predict an increased incidence of dementia or cognitive impairment (12). Shuangling Xiu et al. (13) found that the elderly (community-dwelling participants aged ≥ 55 years) with impaired fasting glucose (IFG) had a lower performance on the MMSE test compared with subjects who had normal blood glucose. Jeannine S Skinner et al. (14) found that high non-diabetic fasting glucose levels were associated with poorer verbal memory and executive function. However, Sai Tian et al. did not find a link between fasting glucose and cognitive impairment (15). Therefore, the relationship between fasting glucose and MCI is not clear, nor can it be determined whether fasting glucose can be developed as a biomarker of MCI.

At present, other advanced biomarkers, such as MRI and PET, for the diagnosis, grading, classification and prognosis of MCI are still under investigation (16, 17). However, there is still little research on serum-based biomarkers that can be easily obtained through minimally invasive sample techniques for diagnosing MCI and assessing treatment response. To fill this gap, in the current cross-sectional study, we explored the practical value of using routine blood and blood biochemical markers, such as fasting glucose, to diagnose MCI.

## 2 Materials and Methods

### 2.1 Participants

Data was obtained from the cohort study on brain health of the elderly in Shanghai (http://www.shanghaibrainagingstudy.org/). This project was launched in 2016, which was a prospective and observational cohort study. The specific content of this project includes understanding the mortality, prevalence, incidence, and population distribution characteristics of mild cognitive impairment and Alzheimer’s disease among the elderly over 55 years old in Shanghai communities. The inclusion criteria were as follows: 1) ≥55 years; 2) permanent population of Shanghai; 3) no evidence of serious mental illness, such as intellectual disability and schizophrenia; 4) no evidence of serious physical illness; 5) agreed to participate in the study. Exclusion criteria were as follows: 1) <55 years old; 2) floating population; 3) serious mental illness and physical illness or acute stress state, for example, acute medical disorders; and 4) the guardians or the participants or refused to participate in the study. Finally, A total of 1015 seniors entered the database, 571 of whom were normal elderly, and the remaining 444 were identified as MCI.

Ethical approval was obtained from the Ethics Committee of the Shanghai Mental Health Center, and all the participants had signed informed consent before the study.

### 2.2 Clinical Evaluations

All the participants would undergo a clinical and cognitive assessment at baseline and follow-up. All the diagnoses were performed by trained and qualified medical clinicians. To improve the accuracy of research, we also collected their body fluids (blood and urine) and head MRI.

#### 2.2.1 Diagnostic Criteria for Mild Cognitive Impairment (MCI)

The diagnosis of MCI was based on Petersen’s diagnostic criteria (18): 1) self-reported or informant cognitive complaints; 2) objective memory disorder; 3) maintain the independence of functional ability; 4) without dementia.

#### 2.2.2 Diagnostic Criteria for the Cognitively Normal Elderly (NC)

All cognitively normal (NC) elderly people needed to meet the following criteria (19): 1) normal cognitive function; 2) without serious physical illness and mental illness; 3) absence of dementia or MCI; 4) be able to complete all tests.

### 2.3 Cognitive Evaluation

In the current study, the Montreal Cognitive Assessment (MoCA) was used to assess the subjects’ overall cognitive function. The MoCA is a brief cognitive screening tool with high specificity and sensitivity for detecting MCI as currently conceptualized in individuals performing in the normal range on the Mini-Mental State Examination (MMSE) (20). Previous studies have shown that a MOCA score of 23, instead of the 26 originally recommended, can reduce the false-positive rate and show better diagnostic accuracy (21). Therefore, in the current study, we also used the MOCA 23 score as the threshold to distinguish MCI from normal.

### 2.4 Blood Routine and Biochemical Tests

Peripheral blood was collected from 7 a.m. to 9 a.m. after overnight fasting. Clot activating gel-containing serum separator tubes were used for testing of blood routine and blood biochemical parameters. During the same time period, samples were taken from each subject at the same approximate time, immediately sent to the hospital laboratory center, and measured before 11 a.m. that day. The red blood cell count, white blood cell count, platelet count, packed cell volume, and hemoglobin were measured by using an analyzer device (Mindray BC-6800, Shenzen, China). The total protein, triglycerides, high density lipoprotein, low density lipoprotein, fasting plasma glucose and glycosylated hemoglobin (HbA1c) were measured by using an analyzer device (Roche Diagnostic COBAS c501).

### 2.5 Covariates

By using standardized questionnaires, we collected general demographic data for these subjects, such as age, education, sex, current smoking status, current drinking status and disease related information, such as diabetes and hypertension. All of these variables would be considered as covariables.

### 2.6 Statistical Analysis

Continuous variables were expressed as mean ± SD and categorical variables were expressed as frequencies (%). Independent sample t-test and Chi-square tests were used to compare the continuous variables and classification variables of the normal group and the MCI group, respectively. Next, we used the binary logistic regression models to examine the association between blood indicators and MCI, treating whether it is MCI or not as a dependent variable. Model 1 did not control any variables; Model 2 controlled some variables, such as education and diabetes. The ROC curve was used to explore the sensitivity and specificity of fasting plasma glucose to predict MCI.

## 3 Results

### 3.1 Comparison of General Demographic Data, Blood Biochemistry, Blood Routine Between the Normal Group and the MCI Group

Compared with the MCI group, the normal group had longer years of education, a higher red blood cell count, a higher hemoglobin number, a higher packed cell volume, and a higher MOCA score, but lower fasting glucose levels and a lower proportion of diabetes(p<0.05). However, there was no statistical difference in age, sex, smoker, drinker, hypertension, white blood cell count, platelet count, hemoglobin, HbA1c, the total protein, triglycerides, high density lipoprotein and low density lipoprotein. **Table 1** presents the results.

**Table 1:** Comparison of general demographic data, blood routine and blood biochemical indexes between the two groups.

Variables	Normal (n = 571)	MCI (n = 444)	X^2^ or t	p
Age, y	69.41 ± 7.535	69.89 ± 7.764	-0.991	0.322
Education,y	11.48 ± 3.482	10.25 ± 3.891	5.320	<0.001*
Male, n (%)	210 (36.8)	140 (31.5)	3.043	0.084
Smoker, n (%)	114 (20.0)	80 (18.0)	0.612	0.469
Drinker, n (%)	99 (17.3)	59 (13.3)	3.117	0.081
Hypertension,n (%)	335 (58.7)	285 (64.2)	3.202	0.080
Diabetes, n (%)	129 (22.6)	125 (28.2)	4.053	0.049*
Red blood cell count, 10^9^/L	4.45 ± 0.597	4.35 ± 0.648	2.229	0.026*
Packed cell volume	0.41 ± 0.054	0.40 ± 0.057	2.311	0.021*
White blood cell count,10^9^/L	6.10 ± 1.568	6.23 ± 1.683	-1.259	0.208
Platelet count,10^9^/L	203.55 ± 57.770	211.05 ± 66.762	-1.809	0.071
Hemoglobin,g/L	133.99 ± 17.665	131.19 ± 19.241	2.275	0.023*
The total protein, g/L	76.47 ± 4.731	76.33 ± 4.406	0.454	0.650
Triglycerides,mmol/L	1.66 ± 1.026	1.73 ± 1.266	-0.908	0.364
High density lipoprotein,mmol/L	1.39 ± 0.374	1.36 ± 0.367	1.179	0.239
Low density lipoprotein,mmol/L	3.10 ± 0.960	3.09 ± 0.964	0.231	0.818
Fasting plasma glucose,mmol/L	5.60 ± 1.378	5.79 ± 1.380	-2.070	0.039*
HbA1c	5.7 ± 0.701	5.8 ± 0.675	-1.038	0.067
MoCA	24.61 ± 2.956	19.19 ± 3.933	25.035	<0.001*

*means p < 0.05; MoCA means Montreal cognitive assessment; HbA1c, means glycated hemoglobin.

### 3.2 Results of Stepwise Binary Logistics Regression Analysis

**Figure 1:**
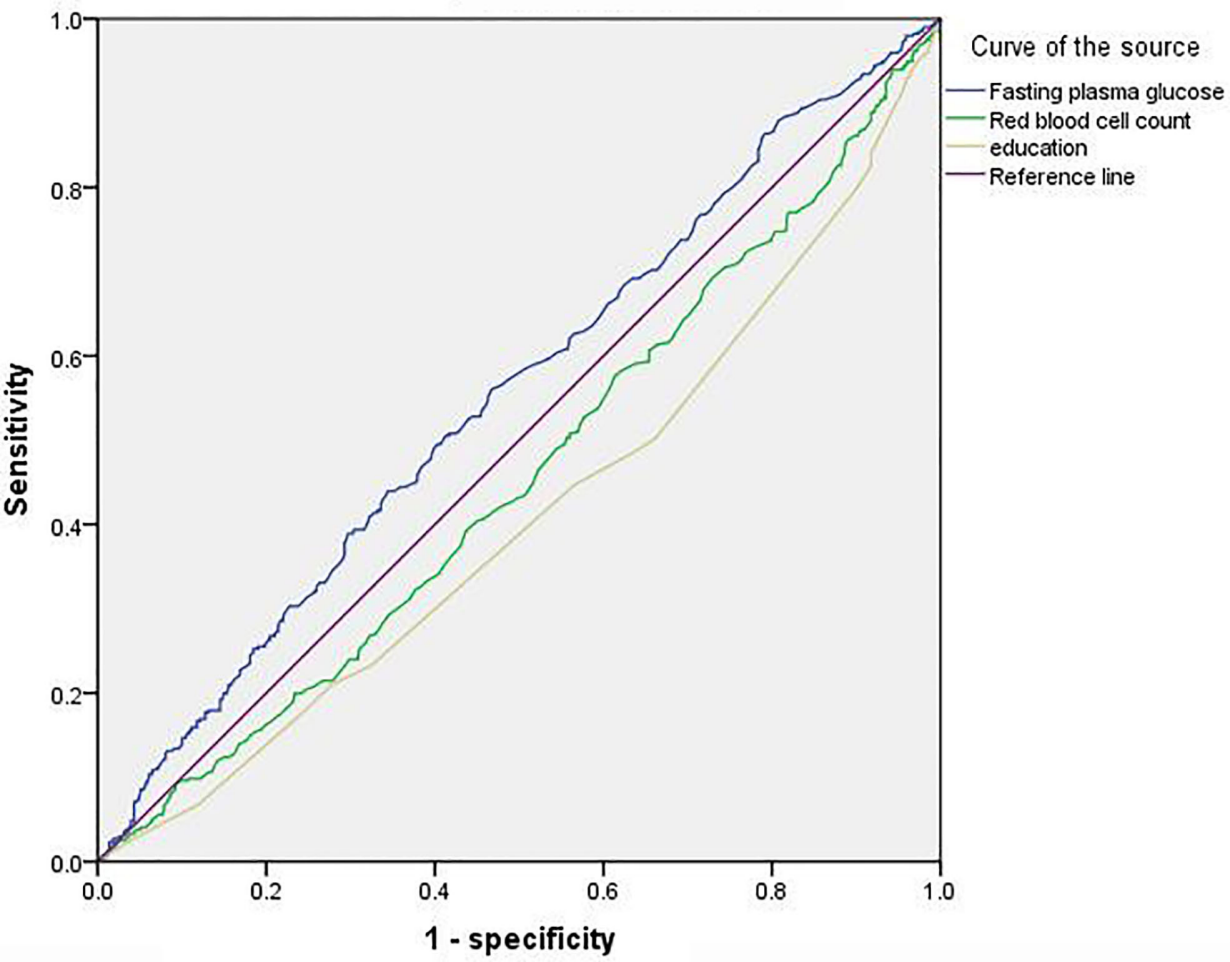
ROC Curve.

**Table 2** shows the results of stepwise binary logistics regression analysis (treating whether it is MCI or not as the dependent variable). Model 1 did not control any covariates (red blood cell count, packed cell volume, hemoglobin and fasting plasma glucose were treated as the independent variables), and the results showed that packed cell volume (p=0.013, OR=0.047, 95%CI:0.004-0.519) and fasting blood glucose (p=0.025, OR=1.118, OR=1.014-1.233) were the influencing factors of MCI. Model 2 built on Model 1 by further controlling for age, education, and diabetes, and did not change the results. **Table 2** shows the results. Then, the ROC curve was used to determine packed cell volume or fasting blood glucose to predict the risk of MCI, and it was found that the area under the curve was 0.457 (p=0.028, 95%CI:0.420~0.495) and 0.553 (p=0.006, 95%CI:0.516-0.591), respectively. And the results suggest that fasting blood glucose had a mild effect in predicting MCI. **Figure 1** presents the results.

**Table 2:** Results of binary logistics regression analysis (with MCI as the dependent variable).

Variables	B	S.E.	Wals	df	p	OR	95% confidence interval
Model 1							
Red blood cell count	-3.057	1.225	6.224	1	0.013	0.047	0.004-0.519
Fasting plasma glucose	0.112	0.050	5.055	1	0.025*	1.118	1.014-1.233
Model 2							
Red blood cell count	-0.298	0.111	7.171	1	0.007*	0.742	0.597-0.923
Fasting plasma glucose	0.119	0.050	5.573	1	0.018*	1.126	1.020-1.243
Education	-0.091	0.019	23.095	1	<0.001*	0.913	0.879-0.947

Model 1 did not control any covariates, and red blood cell count, packed cell volume, hemoglobin and fasting plasma glucose were treated as the independent variables; model 2 further controlled education and diabetes.

## 4 Discussions

In the current study, we explored the relationship between blood routine and common blood biochemical markers and MCI, and ultimately found that elevated fasting glucose was a risk factor for MCI. To my knowledge, this is the first study to demonstrate an inverse association between elevated fasting glucose and MCI. Therefore, we hypothesize that good control of fasting blood glucose might reduce the risk of future cognitive impairment.

Accumulated evidence suggests that abnormal glucose metabolism may contribute to the pathogenesis of MCI, for example, Sai Tian et al. found that increased plasma levels of β-Site APP-cleaving enzyme 1 (BACE1) were associated with poor overall cognition functions, especially visual/logical memory, visuospatial abilities, and executive functions in Diabetes-MCI patients (22). Sai Tian et al. found that high plasma Interleukin-1β (IL-1β) levels were correlated with an increased risk for MCI in diabetes patients (15). Hongjun Zhao et al. found that insulin resistance might be a risk factor for mild cognitive impairment and could be a biomarker for prediction of MCI in patients with diabetes (23). What’s more, our previous work also suggests that diabetes may contribute to the development of MCI (24).

In our study, we explored the relationship between a series of common blood biochemical and routine Indexes and MCI, such as red blood cells, white blood cells, hemoglobin, fasting glucose, etc. Through binary logistics regression analysis, and after controlling several variables such as age and education, we found that red blood cell count and fasting blood glucose were the influencing factors of MCI. However, since the area under the ROC curve of MCI predicted by red blood cell count was less than 0.5, we finally judged that elevated fasting blood glucose was the risk factor of MCI and had a mild predictive effect. Since there was no previous study on the relationship between fasting blood glucose and MCI, we could not judge whether the conclusion of our study was consistent with that of others.

There are several mechanisms to explain why elevated fasting blood glucose increases the risk of MCI. First, poorly controlled fasting blood glucose was associated with the development of long-term micro- and macro-vascular complications (25); Second, elevated fasting blood glucose was strongly associated with depression, which was considered a risk factor for MCI; Third, higher fasting blood glucose levels was associated with decreased striatal and hippocampal volume (26, 27). Fourth, elevated fasting blood glucose was associated with altered N-methyl-d-aspartate receptors/Wnt signaling and oxidative stress in the hippocampus (28). Finally, elevated fasting blood glucose could also lead to elevated inflammatory cytokines (29, 30).

We admit that our research has some limitations. First, it was only a cross-sectional study and could not establish a causal relationship between fasting blood glucose and MCI. Second, we did not further classify MCI, so it is impossible to determine whether fasting blood glucose is more likely to induce amnesiac MCI or vascular MCI.

## 5 Conclusions

Elevated fasting blood glucose may be a risk factor for mild cognitive impairment, but the above conclusions need to be verified by longitudinal studies.

## Data Availability Statement

The original contributions presented in the study are included in the article/supplementary material. Further inquiries can be directed to the corresponding authors.

## Ethics Statement

The studies involving human participants were reviewed and approved by the Ethics Committee of the Shanghai Mental Health Center. The patients/participants provided their written informed consent to participate in this study.

## Author Contributions

WL contributed to the study concept and design. LY acquired the data. SX and LS analyzed the data and drafted the manuscript. All authors contributed to the article and approved the submitted version.

## Funding

This work was supported by grants from the China Ministry of Science and Technology (2009BAI77B03), National Natural Science Foundation of China (number 81671402), Clinical research center project of Shanghai Mental Health Center (CRC2017ZD02), the National Key R&D program of China (2017YFC1310501500), the Cultivation of Multidisciplinary interdisciplinary Project in Shanghai Jiao Tong University (YG2019QNA10), curriculum reform of Medical College of Shanghai Jiao Tong University and the Feixiang Program of Shanghai Mental Health Center(2020-FX-03), Chinese Academy of Sciences (XDA12040101), Shanghai Clinical Research Center for Mental Health (SCRC-MH, 19MC1911100); the National Natural Science Foundation of China (82101564); the Shanghai Science and Technology Committee (20Y11906800).

## Conflict of Interest

The authors declare that the research was conducted in the absence of any commercial or financial relationships that could be construed as a potential conflict of interest.

## Publisher’s Note

All claims expressed in this article are solely those of the authors and do not necessarily represent those of their affiliated organizations, or those of the publisher, the editors and the reviewers. Any product that may be evaluated in this article, or claim that may be made by its manufacturer, is not guaranteed or endorsed by the publisher.
